# Thermomechanical and physicochemical investigation of Raw clay bricks derived from Nomayo clay and palm shell powder lignocellulosic material

**DOI:** 10.1038/s41598-025-13839-x

**Published:** 2025-08-12

**Authors:** Hamka Hamka Adolphe Claudel, Olembe Roland Yves, Djomi Rolland, Ngohe Ekam Paul Salomon, Touani Chualeu Parfait, Biyeme Florent, Tchotang Theodore

**Affiliations:** 1 Civil and Mechanical Engineering Laboratory, École Nationale Supérieure d’Ingénieurs, Université de Yaoundé I, Yaoundé, Cameroon; 2 Energetics laboratory, École nationale supérieure d’ingénieurs, Université de Yaoundé I, Yaoundé, Cameroon

**Keywords:** Unfired clay bricks clay, Palm kernel shell powder, Thermophysical properties, Mechanical strength, Sustainable building materials, Bio-based material, Engineering, Materials science

## Abstract

This study proposes an in-depth characterization of the physicochemical, thermal and mechanical properties of unfired clay bricks derived from Nomayos clay (Cameroon), with progressive additions of palm kernel shell powder bio-based material (0 to 60% by mass). The aim was to assess the influence of lignocellulosic palm kernel shell powder on the physical and mechanical properties of unfired clay bricks, with a view to proposing a sustainable, lightweight, low-cost construction solution. To this end, physical analysis revealed a significant reduction in density (22–35%) with increasing organic matter content, attributed to the lower density of palm kernel shells. Scanning electron microscopy coupled with energy dispersive X-ray spectroscopy (SEM-EDX) confirms a homogeneous distribution of the main elements (C, O, Mg, Fe, Si, Al, Na, K, Ti). X-ray diffraction and Fourier transform infrared spectroscopy analysis show kaolinite as the main mineral phase, with a little amount of quartz and organic functional groups in composite samples. Thermal analysis (TGA/DTA/DTG) indicates a three-stage decomposition process: moisture loss (~ 188 °C), cellulose degradation (~ 314 °C, Δm ≈ 19.17%), and kaolinite dehydroxylation (> 500 °C), with thermal stability reached above 600 °C. Mechanical tests show a progressive decrease in compressive strength from 4.64 MPa (A0%) to 0.12 MPa (A60%), inversely correlated with organic filler content. Despite the decline in mechanical performance, these bricks show potential for lightweight, ecological and low-cost construction.

## Introduction

Humans have used clay resources, mainly for the manufacture of materials and construction, since antiquity. Archaeological excavations have revealed that the Persians, Assyrians, Egyptians, and Babylonians were erecting numerous buildings with this material. Some of these buildings were monumental, such as the Ctesiphon Arch in Iraq, the El-Lahoun pyramid in Egypt, the Etemenanki ziggurat in Babylon, and the pre-Columbian city of Chan Chan in Peru, among many others^1^. Raw earth archaeological sites can be found on every inhabited continent. This is because “earth is one of the three primary materials, alongside stone and wood”^2^. The use of clays in industrial applications depends on knowledge of their physicochemical and technological properties. Clay-based materials are important in the ceramics industry because of their plastic properties and ability to be shaped^3^. With a view to making the most of local materials and solve problems associated with building and heating homes, research into clay bricks incorporating animal, mineral, and vegetable fillers is in full swing. These fillers improve the properties of filled clay bricks, such as resistance to breakage, abrasion, rigidity, abrasive wear, and chemical attack. The loads typically used in mud brick construction (raw and fired) are cement loads. However, observations show that cement makes construction even more expensive. Currently, the most common reinforcing fillers are plant fibers, such as sugarcane and sisal^4,5^. Thus, valorizing agricultural waste is an effective approach to environmental remediation and societal problems. Indeed, numerous studies have examined vegetable fillers, particularly Kenaf fibers^6^ and sugarcane fibers^4^. The results have been verified and found to be satisfactory in terms of both material properties and presentation. For example, Liyong et al. (2024) examined the production of fired clay bricks made from coconut husks and eggshells, creating environmentally friendly compressed earth bricks for sustainable construction. The resulting composite materials were found to be highly durable and thermally stable^7^. Cameroonian clay minerals are widely used for wastewater treatment^8,9^, for the application of Fenton processes^10,11^, for the preparation of zeolites^12–15^, for the depigmentation of crude palm oil^16^, for electrochemical applications^17,18^, and the synthesis of bioenergy from lignocellulosic materials derived from palm oil^19^.

The need to synthesize environmentally-friendly building materials remains a hot topic, as evidenced by its frequent discussion. Replacing this traditional filler with palm kernel shells will be the challenge taken up by construction researchers worldwide, and in Cameroon in particular. Cameroon is a leading palm oil producer in Central Africa. According to the National Institute of Statistics (INS, 2022), the country produced over 2.6 million metric tons of palm oil fruit, generating large amounts of palm kernel shell waste (PKS)^6^. Often incinerated or discarded, these residues offer significant potential for recovery in the construction sector. Several studies have demonstrated that agricultural byproducts, such as sugarcane bagasse, kenaf fibers, and sisal, can improve the thermal insulation and durability of clay bricks while reducing their density. However, despite the abundance of PKS in Cameroon, its use in earth building materials remains largely unexplored, particularly at high incorporation rates and in the context of detailed physicochemical and mechanical analyses. Despite their abundance, palm kernel shells have not been widely studied as fillers in clay bricks. The existing literature mainly focuses on their use in energy recovery (e.g., biochar and activated carbon)^20^ or in the production of lightweight concrete, leaving a significant gap in our knowledge of their impact on the properties of raw earth materials. Furthermore, few studies have conducted a thorough physicochemical, thermal, and mechanical characterization of these composites.

This study aims to address this issue by examining how the addition of palm kernel shell powder affects the properties of unfired clay bricks. The goal is to propose a locally sourced, lightweight, thermally stable, and low-cost material adapted to the needs of palm-growing regions in Cameroon. To this end, formulations containing different proportions of PKS (0–60% by mass) will be prepared. The resulting bricks will undergo in-depth analysis, including density and porosity (physical properties), chemical composition (EDX, FTIR, and XRD), thermal behavior (TGA/DTG/DTA), and compressive strength.

## Experimental materials and methods

### Characterization methods

Equipment used for physical characterization: a mill; a kiln; a pycnometer; a Seditech 1/1000 balance; an AFNOR sieve for particle size analysis; a 1/1000 balance; a USB digital microscope; an automatic mixer; a vibrating table for compacting bricks^21^; for chemical characterization: (XRD, FTIR, SEM, EDX-MAPPING), a Bruker Alpha spectrometer with the ATR (Attenuated Total Reflection) technique on a diamond crystal. Resolution during spectra collection is set at 4 cm^− 1^, from the laboratory of Inorganic Chemistry, University of Dschang (Cameroon)^22^, Structural identity of all samples was determined by X-ray diffraction (XRD) using an Empyrean powder diffractometer (PANanalytical, Almelo, Netherlands) equipped with a Cu tube (wavelengths Kα1 = 1.540598 Å and Kα2 = 1. 544426 Å) at 40 mA and 40 kV. All samples were scanned from 4 to 90 at 2θ°, morphology and microstructure of all samples were analyzed by FEI SEM XL30 field emission scanning electron microscope (FEI, Hillsboro, USA) equipped with energy dispersive X-ray spectrometer at 20 kV accelerating voltage; for thermal characterizations: Thermogravimetric analysis (TGA) and differential thermal analysis (DTA) were carried out using a Netzsch STA 449 C Jupiter thermogravimetric analyzer (Netzsch, Selb, Germany); for mechanical characterizations: We used an Impact Test Equipment Limited electrohydraulic flexure-compression testing machine, 250 KN, with a plate descent speed set at 0.5 MPa/s. For each formulation, the strength obtained is the average of tests performed on three 40 × 40 × 40 mm cubic specimens^23^.

### Materials

The clay used in this study was collected from two strategic sites in Cameroon: lateritic clay from the rural area of Noyamos I (3°28’N, 11°16’E) in the commune of Mbankomo (Méfou-et-Akono department, Centre region), selected for its optimum ceramic properties (58 ± 3% kaolinite content, 17 ± 2 plasticity index), and palm kernel shells from the urban area of Souza (4°14’02 “N, 9°36’48 “E) in the commune of Bonaléa (Littoral region). Noyamos clay was chosen for its well-documented kaolinitic properties and plasticity^24,25^ while Souza palm kernel shells represent a readily available agricultural by-product in this palm-growing region of Central Africa^25^.

### Elaboration of clay-shell powder mixtures and manufacture of test specimens

**Fig. 1:**
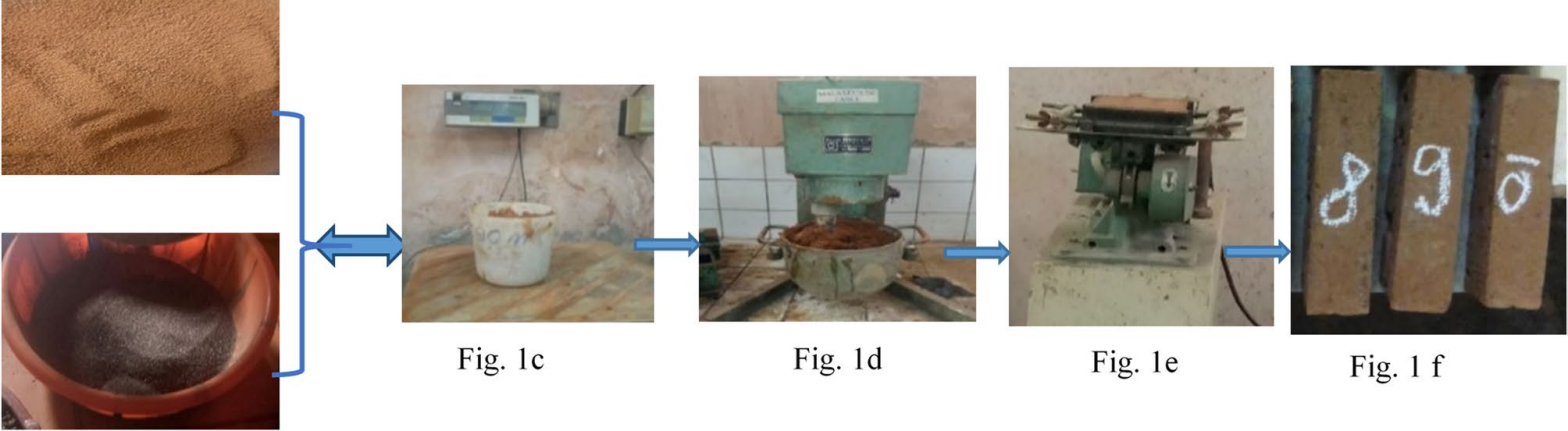
Process for obtaining raw clay bricks filled with palm kernel shell powder.

Figure 1 illustrates the experimental procedure used; Fig. 1a and b show the clay and shell powder obtained at the desired particle sizes, respectively, then Fig. 1c, d, e and f describe the process of obtaining clay bricks in compliance with predefined dosages, ranging from 0%, 5%, 15%, 25%, 35%, 50% and 60% of the shell powder charge in the clay matrix. The mixtures were prepared according to Eq. (1)^21^.

1 $$\:\left\{\begin{array}{c}{M}_{f}=\frac{{\rho\:}_{f}}{{\rho\:}_{c}}{V}_{f}\:\Rightarrow\:{M}_{f}=\frac{{\rho\:}_{f}}{{\rho\:}_{f}\text{.}{\text{V}}_{f}+{\rho\:}_{m}\left(1o{V}_{f}\right)\:}{V}_{f}\text{}\text{}\text{}\\\:{M}_{m}=\frac{{\rho\:}_{m}}{{\rho\:}_{c}}{V}_{m}\:\Rightarrow\:{M}_{m}=\frac{{\rho\:}_{m}}{{\rho\:}_{f}.{V}_{f}+{\rho\:}_{m}\left(1\:{V}_{f}\right)}{V}_{m}\text{}\text{}\text{}\text{}\text{}\text{}\text{}\text{}\end{array}\right.$$

The masses of clay and palm kernel shell powder were accurately measured (Fig. 1c) and poured into the mixer (Fig. 1d). Mixing was carried out (Fig. 1d) in the geotechnical and materials laboratories of the Ecole Nationale Supérieure Polytechnique de Yaoundé (ENSPY), using an automatic table-top mixer manufactured by Maurice PERRIER et Cie, 20 rue Marie Debos 92,120. MONTROUGE (France), Type 32, N0 651, to completely homogenize the paste ready for brick production. The clay and palm kernel shell mortar is mixed and kneaded, to get as close as possible to the model of a homogeneous, isotropic material, until clay bricks are obtained (Fig. 1f) for possible characterization^21^.

### Experimental methodology

#### Mechanical treatment of study materials

Mechanical treatment was carried out in the laboratories of Mipromalo (Mission de Promotion des Matériaux Locaux). Particle size analysis by dry sieving after washing was performed according to standard NF P 94 − 056^21^. Particle size analysis by sedimentometry² was carried out in accordance with standard NF P 94 − 057, following the procedure described in the literature^21^. Methylene blue tests were carried out in the Mipromalo laboratories on clay samples in accordance with standard NF P 94 − 068^21^. Atterberg limits were determined in two separate phases in accordance with standard NF P 94 − 051^26^. For water content, we weighed the vacuum tare previously cleaned and dried in the oven; we put a quantity of material in the tare and weighed the wet mass; then the material was introduced into the oven and put at 105 °C for 24 h, after which the dry mass of the material was weighed; the water content (EC) of the treated clay soil was determined by Eq. (2) below.

2 $$\:{ TE\left(\%\right)}^{}=\frac{\left(Mh-Ms\right)}{Ms}x100$$

where **TE**: water content, **Mh**: wet mass and Ms: dry mass.

To determine the true density, first, the empty pycnometer was weighed. Then, the pycnometer was filled with distilled water up to the gauge line, and its weight was recorded. Next, a quantity of distilled water (more than half) was removed, and 20 g of the sample was introduced into the pycnometer. Finally, the pycnometer was placed on a hot plate and heated to boiling. After cooling the mixture in air, then in a water bath, a quantity of water was added up to the calibration mark, and the mixture was weighed^21^; the true density (ρ) of the treated clay was determined using Eq. (3) below.

3 $$dr=\frac{(M2-M1)}{(M2-M1)-(M3-M4)}$$

With M1: mass of empty pycnometer, M2: mass of pycnometer with sample, M3: mass of pycnometer, sample and water, M4: mass of pycnometer with water and dr: actual density.

#### Determination of properties

The moisture content, bulk density (wt), saturated density and water absorption capacity of the shell powder were determined according to the methodology of Djomi et al. (2018)^27^. Brick bulk density was calculated using standard methodology in accordance with ASTM D7348–2008, with reference to the experimental framework of Pape Moussa Toure^28^. The methodology for chemical and thermal characterization is the same as that described for clay analysis. Compressive strength tests were carried out according to ASTM D2344/D2344M^29^, and flexural strength tests followed ASTM D790. A 250 kN electrohydraulic universal testing machine (Impact Test Equipment Ltd.) was used, with a constant crosshead speed of 0.5 MPa/s^30^. For each formulation, the strength obtained is the average of tests performed on three 40 × 40 × 40 mm cubic specimens.

## Results and discussion

### Physical characterization of green clay bricks

#### Bulk density

**Fig. 2:**
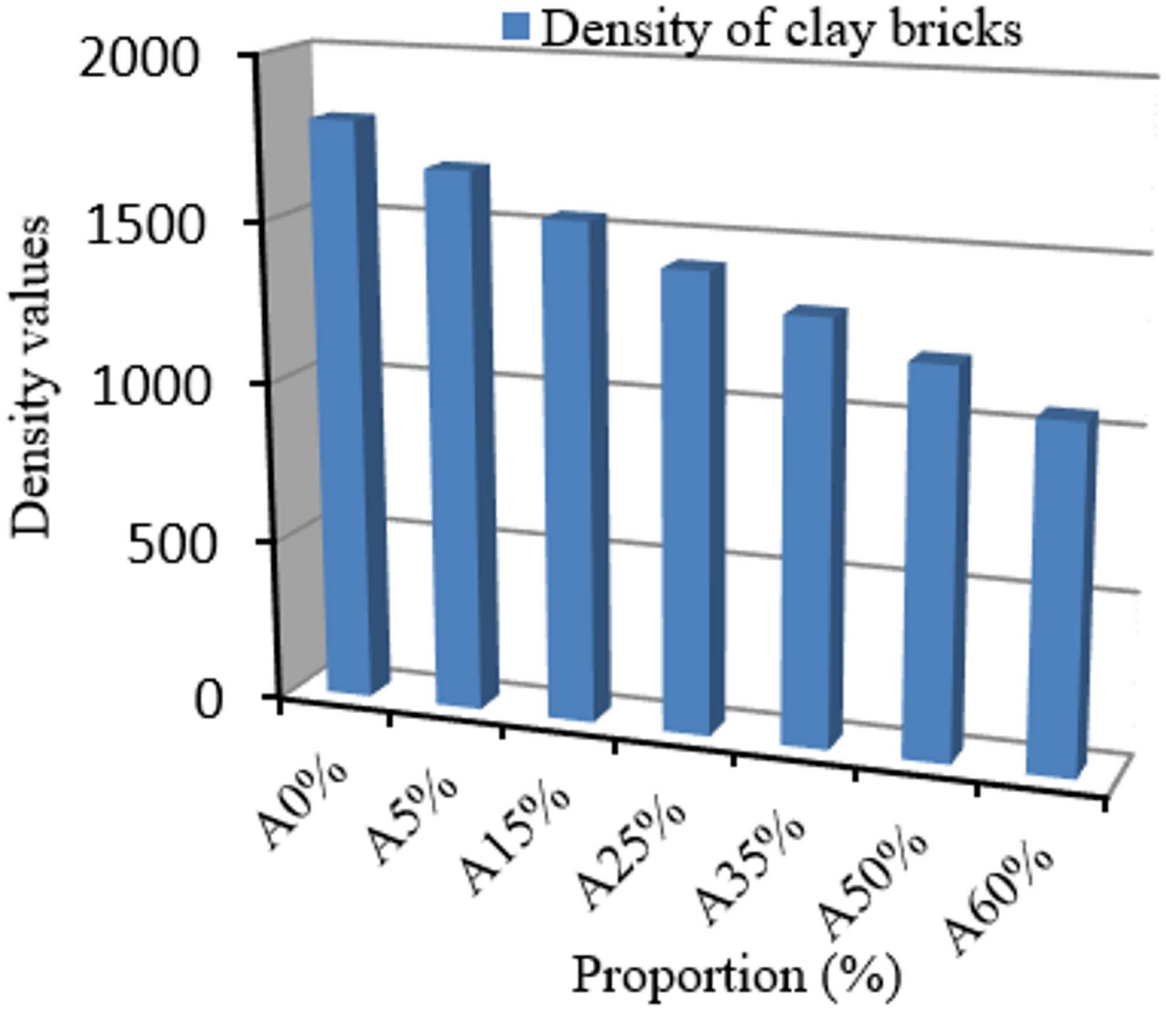
Density results for green bricks filled with palm kernel shell powder.

Figure 2 shows the results for the bulk density of the manufactured bricks. The data in this figure reveal a significant reduction in density from 1801.45 kg/m^2^ for unfilled clay bricks to 1060.73 kg/m^2^ for composites containing 60% palm kernel shell powder. This result can be explained by the substantial difference between the densities of the constituent materials: 1.85 g/cm^2^ for Noyamos clay versus 0.689 g/cm^2^ for PKSP. The light palm kernel shell powder effectively occupies the interstices of the clay matrix, creating a porous structure that reduces overall mass while maintaining structural integrity. From this result on the density of the material, we can therefore confirm that it could be ideal for high-rise constructions due to its lightness. These results are similar to those obtained by Belhadj Ali et al. (2018)^31^, but lower than those reported by Oti et al. (2017)^32^ for concrete reinforced with walnut shell powder (1562–2042 kg/m^2^). This confirms the lightweight effect of clay filled with palm kernel shell powder.

#### Actual density and porosity

**Fig. 3:**
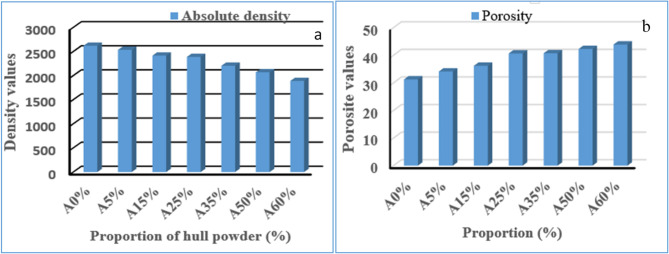
Actual density (**a**) and Porosity (**b**) results.

Figure 3a shows the actual density results for the bricks, while Fig. 3b presents the porosity results. In addition to bulk density, the porosity of each brick formulation was estimated using the relationship between bulk density and true density, as defined in ASTM C20-00^33^:

$${\text{Porosity}} (\%) =1-({\text{Apparent \, density/Actual \, density}})1-({\text{Apparent \, density/Actual \, density}})1-({\text{Apparent \, density/Actual \, density}}) \times 100.$$

.

The data presented in Fig. 3 reveal a significant reduction in density, from 2620.28 kg/m³ for A0% formulations to 1890.32 kg/m³ for A60% formulations. On the other hand, the study of porosity reveals a gradual increase as a function of the quantity of palm kernel shell powder: from 31.25% for the A0% formulation to 42.72% for A60%. This trend is due to the lower intrinsic density of hull powder compared to clay, which increases the volume of voids in the material. A stabilization of porosity beyond 25% of the proportion of shell powder in the material is observed, reflecting a saturation phenomenon within the porous structure. These observations are consistent with an observed reduction in compressive strength. Numerous studies show that the incorporation of organic materials such as palm kernel shell powder into the material promotes an increase in porosity. According to Aeslina et al. (2017)^34^, fired bricks containing between 1 and 10% palm kernel shell powder display porosities of 16–22%. Similarly, Githinji et al. (2016)^35^, found porosities ranging from 28 to 38% when adding biomass. Alengaram et al. (2022)^36^ report values ranging from 22 to 35% when using a mixture of palm kernel shell powder and clay. In comparison, our values (31–43%) are higher and can be attributed to the absence of firing and a hydraulic binder.

### Chemical characterization of bricks

Advanced characterization techniques are required to assess the suitability of clay-palm kernel shell composites for building materials. X-ray diffraction (XRD) identifies the crystalline phases in the clay and monitors phase changes due to heating or chemical modification. Fourier transform infrared (FTIR) spectroscopy identifies functional groups and detects chemical bonds and interactions between the clay matrix and organic fillers. Energy dispersive X-ray spectroscopy (EDX), when combined with scanning electron microscopy (SEM), provides quantitative information on the spatial distribution of elemental composition. This confirms the incorporation of organic matter and analyzes the purity of clay minerals. Together, these techniques help us understand structure-property relationships and optimize the thermal and mechanical performance of the final material.

#### FTIR results

**Fig. 4:**
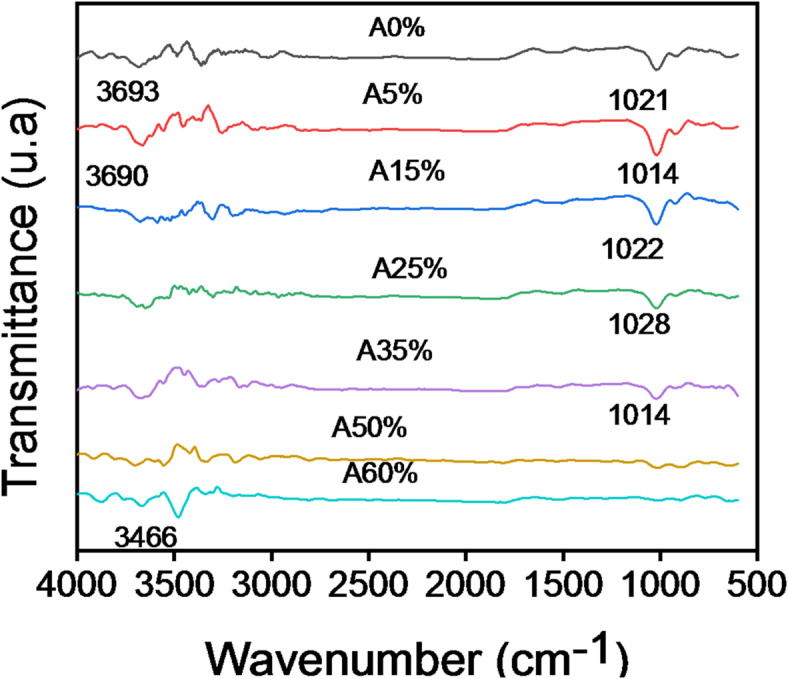
FTIR spectra of raw bricks filled with palm kernel shell powder^39^.

Figure 4 shows the Fourier transform infrared spectra of unfired clay bricks that incorporate palm kernel shell powder (PKSP) at defined dosage levels (0%, 5%, 15%, 25%, 35%, 50%, and 60%). The unmodified clay brick (A0%) exhibits an intense band at 1021.123 cm^− 1^, which is attributed to the Si-O-Si antisymmetric stretching vibration of kaolinite or quartz tetrahedral structures^22^. In samples containing PKSP (A5%-A35%), this band shifts slightly to lower wavenumbers (1014.24–1020.08 cm^− 1^), which confirms interaction between the clay matrix and the organic components. Similar vibrational shifts have been reported in other clay-biomass composite systems^25,37,38^.

The broad absorption between 3620 and 3695 cm^−1^, visible in all samples, corresponds to the stretching vibrations of the hydroxyl groups of kaolinite and intercalary water. The band observed at 3693.014 cm^−1^ in the A0% sample and maintained in the A5%-A50% samples suggests a stable clay matrix structure. This vibration becomes more intense in the A60% sample, likely due to the cellulose-derived -OH groups introduced by PKSP^29^. Other absorption peaks at 915 and 750–790 cm^−1^ are linked to Al-OH bending, which is typical of kaolinitic minerals^22^. Additional peaks in the PKSP samples, ranging from 2850 to 2950 cm^−1^, indicate aliphatic C–H stretching. A shoulder at approximately 1735 cm^−1^ suggests C = O stretching vibrations, which are commonly attributed to lignin and hemicellulose residues^37,38^. A band close to 1625 cm^−1^ may correspond to adsorbed water or aromatic C = C bonds in residual organic matter. Overall, these FTIR features confirm the presence of palm kernel shell powder components and their molecular integration into the clay matrix. This provides a spectroscopic basis for the observed physical and thermal changes. A small shift towards higher allande wavenumbers, from 1021 to 1028 cm^−1^, can be observed with an increase in palm kernel shell powder in the material, which can be explained by: Changes in the local chemical environment around the Si and Al atoms linked to the introduction of organic matter and a decrease in the degree of local crystallinity in phyllosilicates.

#### XRD results

**Fig. 5:**
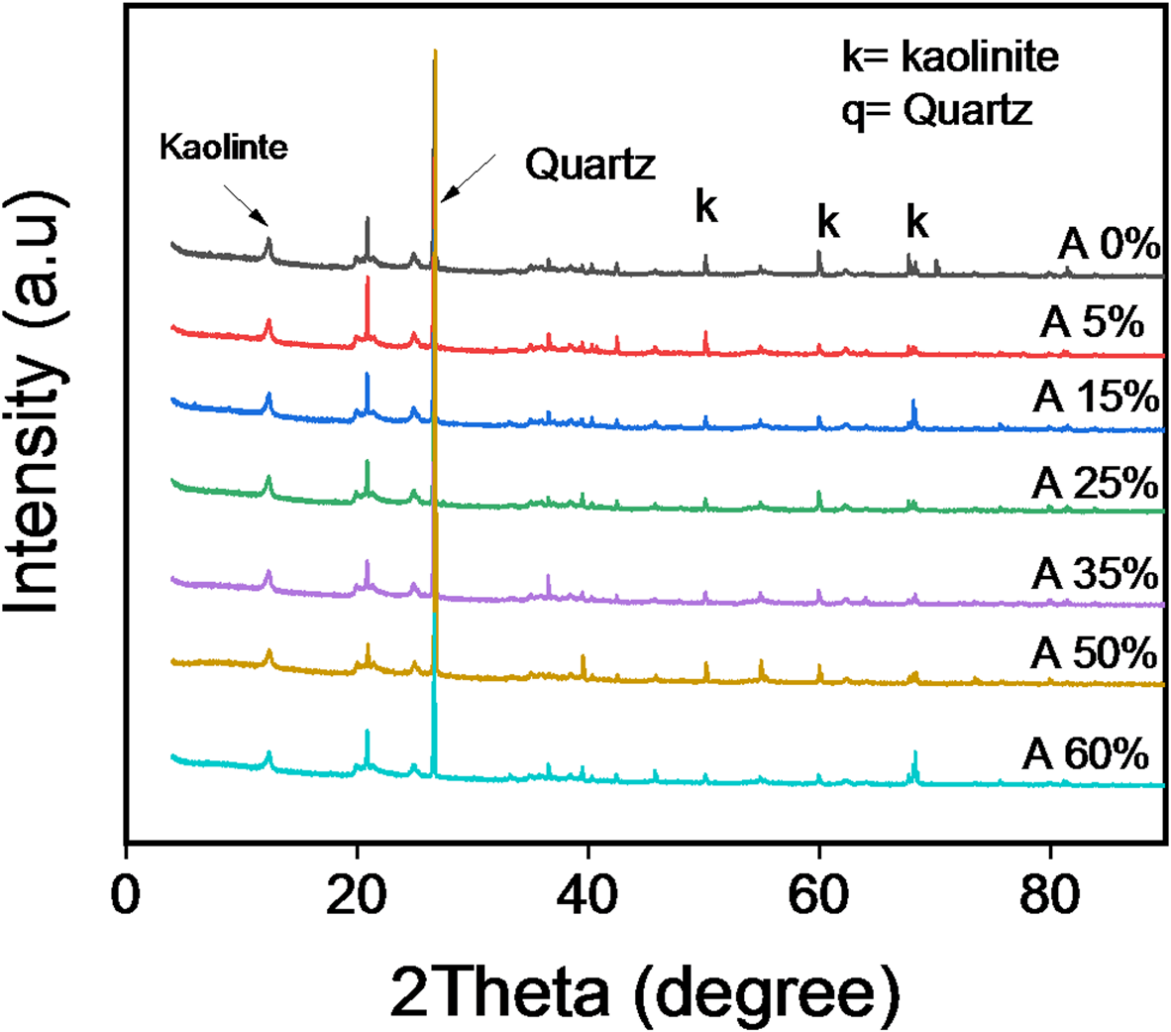
XRD pattern results for clay bricks loaded with palm kernel shell powder.

Figure 5 shows the XRD pattern of clay bricks from unfilled composites (A0%) and composites based on palm kernel shell powder (PKSP) with loading levels ranging from 5 to 60%. The A0% sample shows characteristic kaolinite reflections at 2θ ≈ 10° and 20° (d-spacing = 7.31 Å, calculated using Bragg’s law)^40^ and Quartz visble at 2θ ≈ 23°. XRD pattern for the A0% formulation reveal sharp, intense peaks, confirming the strong crystallinity of pure clay and the dominant presence of kaolinite. These kaolinite signatures persist in all samples loaded with palm kernel shell powder (A5%-A50%), demonstrating the structural stability of this 1:1 phyllosilicate due to its strong interlayer hydrogen bonding and minimal interfoliar space. This space remains intact despite the incorporation of organics. Conversely, in the A5%-A25% formulations, slight attenuation of the peaks is observed, which may indicate the beginning of crystal lattice disruption. Between A35% and A60%, the peaks broaden and weaken due to a marked reduction in crystallinity. Overall, the results obtained confirm that the gradual introduction of palm kernel shell powder into the material leads to a gradual decrease in crystallinity, especially from the A35% formulation; also, no extraneous diffraction signals the formation of new phases, but rather indicates structural disruption. Work similar to that of Sutcu et al. (2015) shows that the addition of agricultural waste (straw, rice husks) to clay reduces overall crystallinity, while retaining the main phases.

#### SEM results

**Fig. 6:**
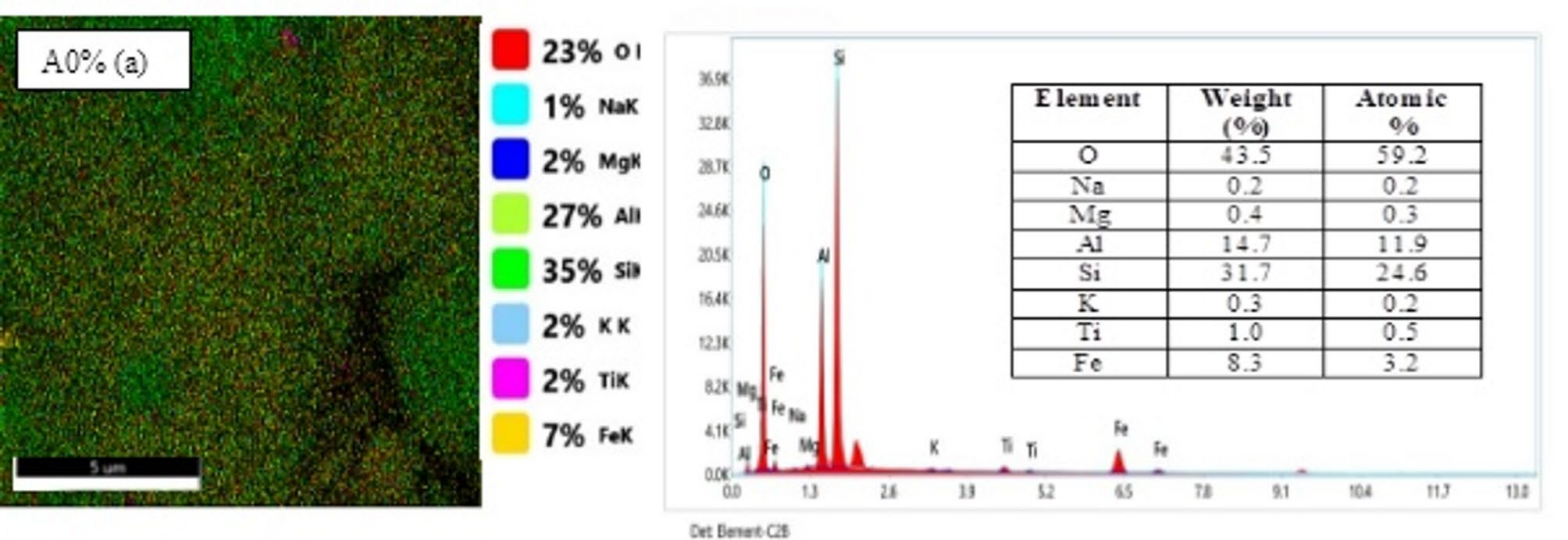
SEM images for green bricks loaded with palm kernel shell powder.

The scanning electron microscope analysis in Fig. 6 illustrates the results obtained for various clay brick compositions incorporating increasing proportions of palm kernel shell powder. Formulation A0% (unfilled clay) has a rough texture with uniformly distributed medium-sized particles^41^. However, formulations A5%, A15%, A25%, A35% and A60% show a progressive agglomeration of particles proportional to the organic matter content, with the exception of sample A50% which shows an intermediate morphology with grain uniformity partially restored thanks to a balanced distribution between clay and palm kernel shell powder^42^. The porous surface morphology, characterized by randomly oriented platelets, confirms the suitability for ceramic application^27^, with EDX analysis identifying the fundamental components of the clay (Si-tetrahedral, Mg/Al-octahedral centers, O-layers and Ti/Fe/K intermediate layers) and revealing carbon signatures in the doped samples that systematically intensify with palm kernel shell powder (PKSP) loading, verifying the success of organic incorporation through a clay brick.

#### EDX and EDX mapping results

**Fig. 7:**
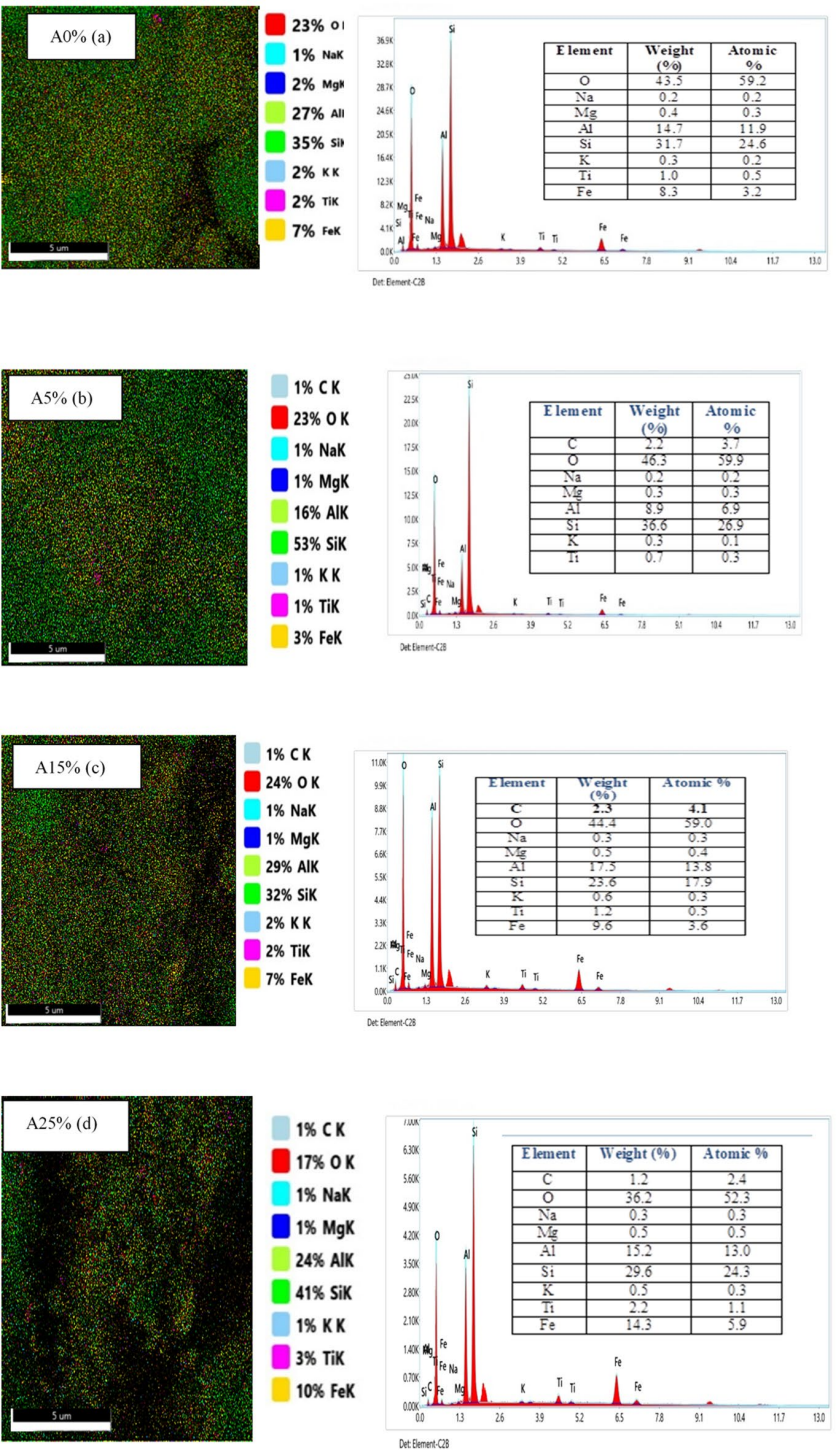

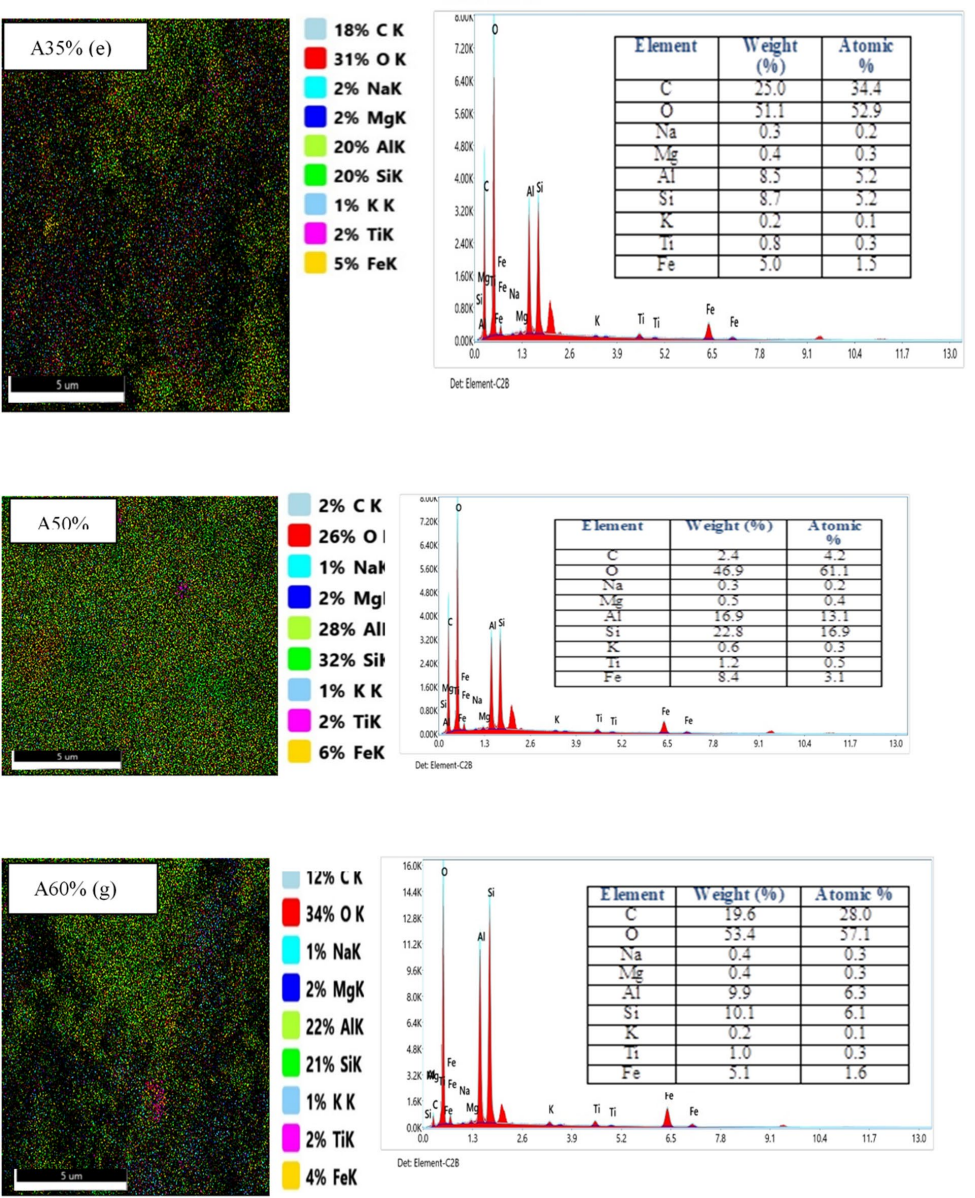
EDX mapping results for green bricks loaded with palm kernel shell powder.

**Fig. 8:**
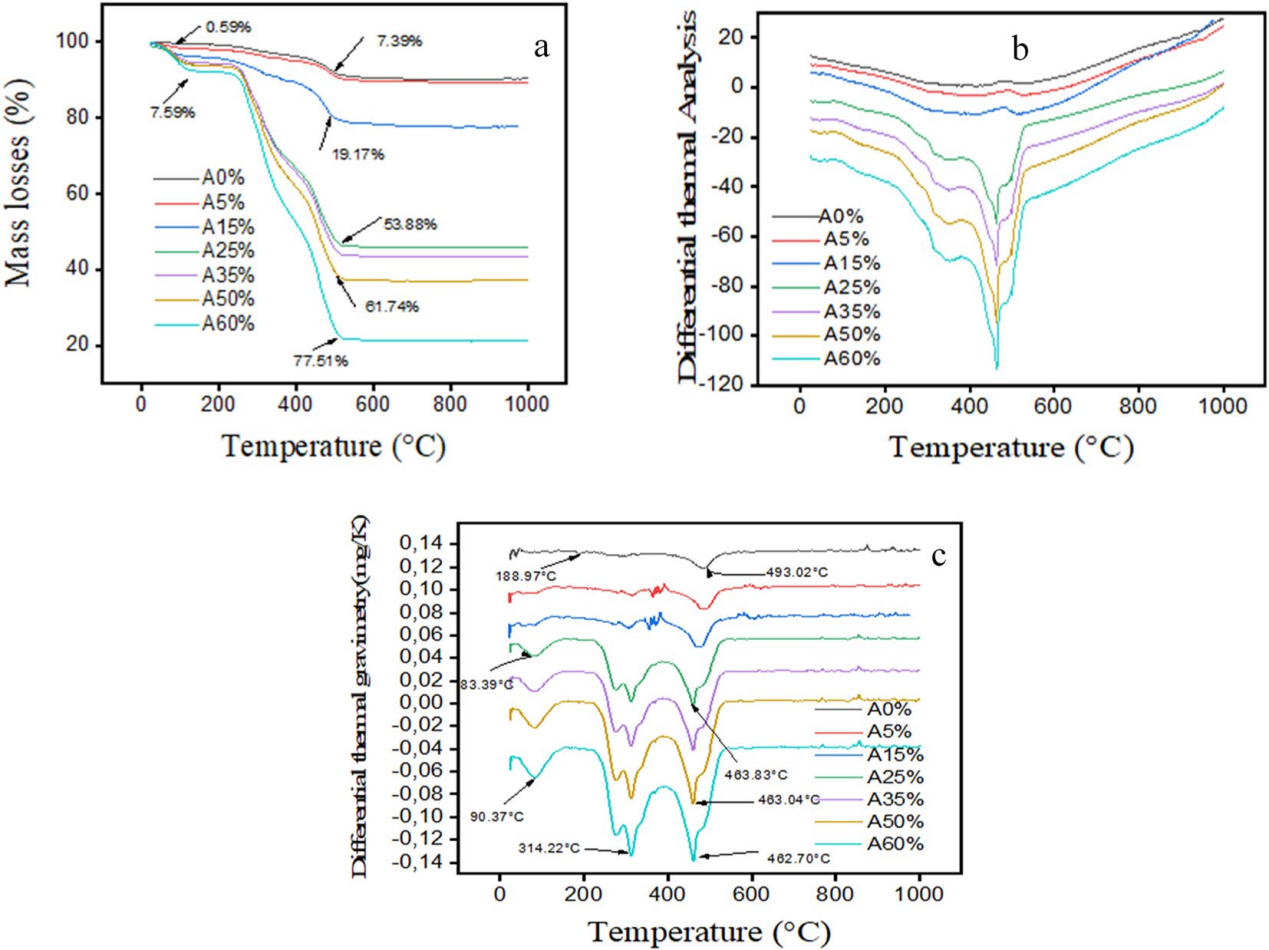
TGA (**a**), DTG (**b**) and DTA (**c**) analysis for clay and composite bricks incorporating palm kernel shell powder.

EDX analysis for samples A0%, A5%, A15%, A25%, A35%, A50% and A60% are shown in Fig. 7, revealing the different atoms present in each material. Figure 7A0% (a) corresponds to untreated clay. It reveals the presence of several chemical elements (O, Mg, Fe, Si, Al, Na, K and Ti). The mass and atomic concentration of each element are recorded in a table accompanying the spectrum of each material. The presence of the elements Al, Si and O in greater quantities can be attributed to the structural nature of the clay matrix, which is mainly composed of aluminum octahedra and silicic tetrahedra. However, the presence of trace amounts of potassium (K) (0.3% by mass and 0.2% by atoms) can be explained by its incomplete exchange with Na^+^ ions in the deeper layers during the homionic saturation process [9]. With the exception of Fig. 7a, the spectra of the other Figs. 8 (A5% - A60%) reveal the presence of all the chemical elements identified in the clay sample (C, O, Mg, Fe, Si, Al, Na, K and Ti). Additionally, we observed the appearance of a new chemical element, carbon (C), which explains the addition of palm nut shell powder to the composite at the indicated percentages. Furthermore, sample A60% has a high carbon concentration, with a mass percentage of 19.6% and an atomic percentage of 28.0%. This is due to the high proportion of palm kernel shells in this sample compared to the others. These observations confirm the formation of a composite brick. Futhermore, the associated EDX mappings reveal a discontinuous and heterogeneous distribution of elements (C, O, Mg, Fe, Si, Al, Na, K and Ti), suggesting the formation of a composite brick with strong adhesion between walnut shells and clay. This is probably due to the bonding between the hydrolyzed walnut shells and the AlO₄/SiO₄ tetrahedra of the clay. Overall, the EDX images reveal a uniform microstructure with a low concentration of palm kernel shell powder ranging from 0 to 25%. Once the addition of hull powder exceeds 35%, the map reveals increased heterogeneity, suggesting an uneven distribution of elements, particularly carbon and calcium. This progression affects the mineralogical composition of the bricks. Additionally, the morphology reveals a scattered granular texture with zones loaded with organic matter (high carbon concentration). This variability could impact mechanical and thermal properties, such as reduced conductivity and increased porosity. A similar result was found to those of Elimbi et al.^43^.

#### AGT results

The results of thermogravimetric analysis (TGA) and differential thermal analysis (DTA) are shown in Fig. 8.

The first mass loss (0.59%) observed in the A0% sample at around 200 °C corresponds to the removal of water physically adsorbed by the clay, as shown by the TGA curve (Fig. 8a). This is also confirmed by an endothermic peak at 188.97 °C in the DTG curve (Fig. 8c), which aligns with the TGA/DTA data. A second endothermic event occurs at 493.02 °C, marked by a peak in the DTG curve (Fig. 8a) and a mass loss of 7.39% in the TGA curve (Fig. 8a) for sample A0%. This corresponds to the dehydroxylation of kaolinite, in agreement with the following reaction (b.4):

$$\:Al2Si2O5\left(OH\right)4\:\to\:\:Al2O32SiO2\:+\:2H2O$$

4 $$\:Al2O32SiO2\:\to\:\:\frac{1}{3\left(3AL2O32SiO2\right)}\:+\:\frac{4}{3\left(SiO2\right)}$$

This loss of mass corresponds to the evolution of water physically adsorbed from the lignocellulosic structure, confirming both the crystalline nature of the palm kernel shells and their successful integration into the clay matrix, as shown by the reproducible thermal decomposition profile. Furthermore, in samples with a higher palm kernel shell content (A25%-A60%), the DTG curve (Fig. 8c) shows a pronounced endothermic peak at 314.22 °C, accompanied by a mass loss of 19.17% in the TGA data (Fig. 8a). This thermal event corresponds to the cellulose-lignin transition phase^44,45^, indicating the substantial energy input required for cellulose decomposition, due to the greater mass loss for the 50% and 60% loaded composites Above 600 °C, the system reaches a thermodynamic equilibrium characterized by: (i) complete conversion to metakaolin in A0% (amorphous phase confirmed by XRD), and (ii) complete lignocellulosic decomposition in composites (A5%-A60%), leaving only mineral ash constituents.

### Mechanical characterization results

#### Compressive strength results

**Fig. 9:**
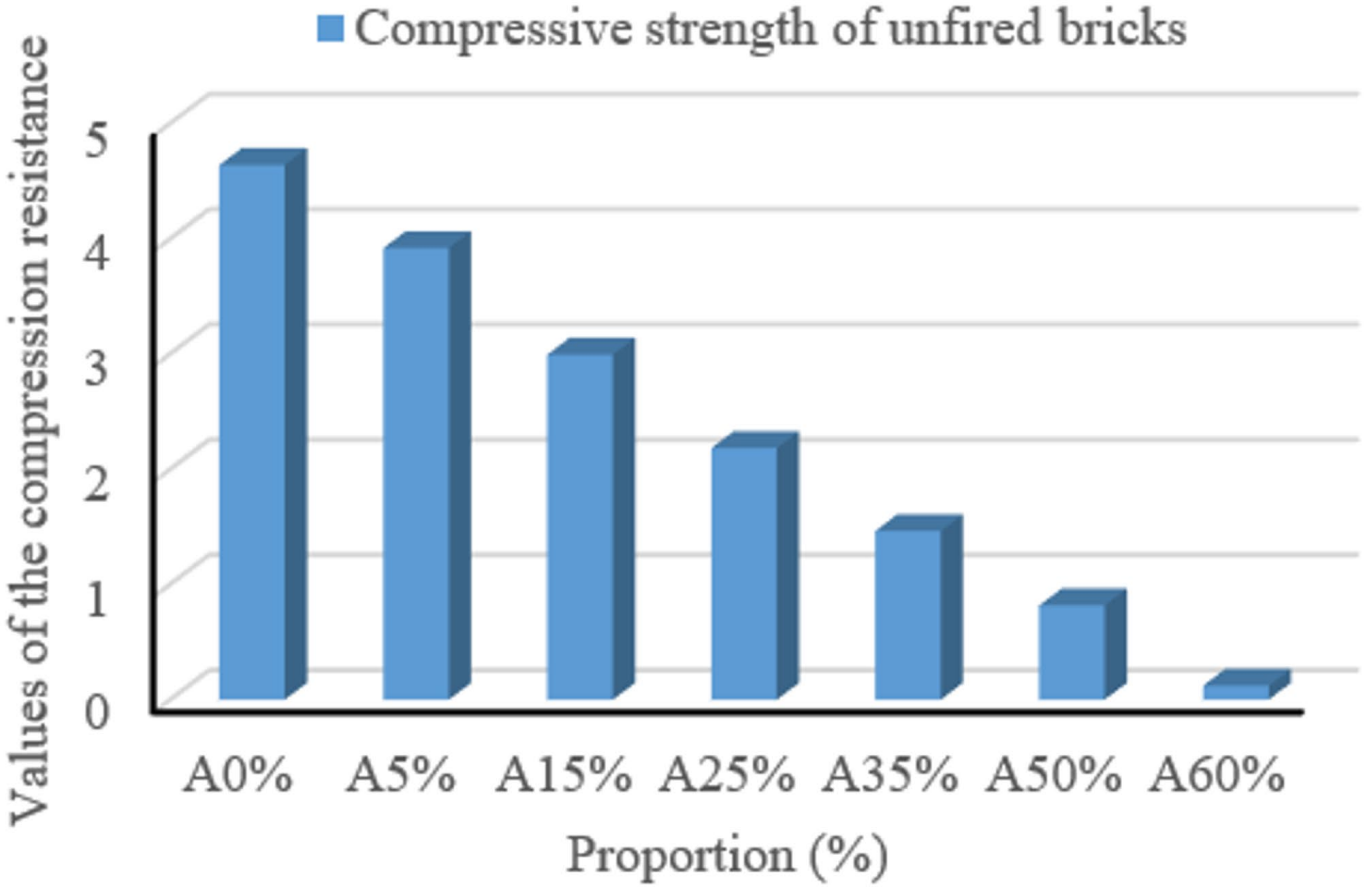
Compressive strength of mud bricks filled with palm kernel shell powder.

Compressive strength results for earth bricks incorporating palm kernel shell powder at different dosages (A0%-A60%) are shown in Fig. 9. The strength ranges from 4.64 MPa for the control sample (A0%) to 0.12 MPa for the A60% sample. Clearly, increasing the palm kernel shell powder content significantly reduces the bricks’ compressive strength. This reduction is attributed to organic particles that disrupt the compact structure normally provided by the clay matrix. These results differ greatly from those reported by Anagonou et al. and Riyap et al.^23^. This comprehensive study systematically characterized unfired clay bricks incorporating palm kernel shell powder (0–60% by weight), revealing three key results: (1) compressive strength decreases proportionally to bio-aggregate content (4.64→0.12 MPa), attributed to reduced structural compactness; (2) bulk density decreases significantly, improving lightweight properties ideal for multi-storey foundations; and (3) multi-technique analyses (FTIR/TGA-DTA/SEM-EDX) confirm stable thermal behavior (> 300 °C) and compatible clay-powder interfaces. Although mechanical performance decreases, the material offers convincing advantages in terms of sustainability - use of local agricultural waste, reduced embodied energy and improved thermal insulation - making it technically viable for environmentally-friendly construction in palm-growing regions, even if it differs from some literature values^23^.

#### Summary of physico-thermo-mechanical properties and comparative discussion paragraph

Table 1 summarizes the bulk density, true density, calculated porosity, thermal degradation behavior via TGA, DTA, DTG and compressive strength of the samples, highlighting the impact of increasing PKSP content on material performance.

**Table 1:** Summary of physical, thermal and mechanical properties.

Formulation	Density (kg/m^2^)	Compressive strength (MPa)	Mass lossTGA (%)	DTA peak (°C)	DTG peak (°C)	Estimated actual density (kg/m^2^)	Porosity (%)
A0%	1801.45	4.64	7.59	493.02	189–494	2620.28	31.25
A5%	1670.06	3.91	7.39	470	83.86	2535.15	34.12
A15%	1541.42	2.99	19.17	470	90.53	2415.12	36.18
A25%	1417.84	2.18	53.89	465	313.26/463.83	2389.04	40.65
A35%	1307.16	1.46	61.71	465	312.89/463.04	2205.25	40.73
A50%	1194.99	0.81	77.54	460	313.04/463.04	2070.05	42.27
A60%	1060.73	0.12	80.03	460	314.22/462.70	1890.32	43.89

Compared with similar studies, the present results confirm well-known trends and reveal characteristics specific to the use of palm kernel shell powder (PKSP). For instance, Michael and Moussa (2021) observed a 28% reduction in brick density with sugarcane bagasse reinforcement, which is similar to the 34% reduction observed here with 60% PKSP reinforcement. In their study, compressive strength dropped from 4.90 MPa to 0.85 MPa as fiber content increased, which is consistent with the degradation observed in our samples (4.64 MPa to 0.12 MPa). In another study, Akinwande et al. (2024) reported strength values ranging from 3.6 to 1.2 MPa in lateritic bricks reinforced with kenaf fibers and observed a similar inverse correlation between strength and fiber content. The current study further demonstrates that the kaolinitic matrix interacts chemically with PKSP residues. This interaction is evident through thermal degradation and FTIR peaks. This provides unique insight compared to studies using inert fillers, such as quarry dust.

These comparisons highlight that, although PKSP reduces mechanical strength, it provides better thermal behavior and contributes to material lightening, which is particularly useful in non-load-bearing applications such as interior partitions in tropical climates. The incorporation of palm kernel shell powder (PKS) influences the physical, mechanical, and thermal characteristics of raw clay bricks. The results are compared with those documented in the scientific literature to evaluate the significance of the identified trends. At first glance, the bulk density increased from 1,801.45 kg/m^2^ (A0%) to 1,060.73 kg/m^2^ (A60%), while the true density decreased from 2,620.28 kg/m^2^ to 1,890.32 kg/m^2^. This is due to the low intrinsic density of the powder, which partially replaces the heavier mineral grains. Similar decreases have been observed in comparable research (Ismail et al., 2019; Sutcu et al., 2015) when integrating light biomasses, such as rice bagasse or sawdust. Second, compressive strength decreased from 4.64 MPa (A0%) to 0.12 MPa (A60%). This reduction is attributed to weakening of the bond between particles resulting from increased porosity and thermal decomposition of the organic material. Elimbi et al. (2011) recommend a minimum strength of 2 MPa for green bricks. Muñoz et al. (2020) found that excessive incorporation of plant fibers (> 40%) considerably reduces mechanical strength. Additionally, TGA observation reveals a significant increase in mass loss, from 7.59 to 80.03%. This indicates that the organic fraction increases with the addition of palm kernel shell powder. DTA peaks show a slight decrease (from 493 °C to 460 °C), indicating a reduction in thermal stability. The DTG curves change from a single peak to two distinct peaks when the composite reaches 25% hull addition, which corresponds to the dehydration and decomposition of certain organic components.

These findings are consistent with Sutcu et al.‘s results for lignocellulosic materials^46^. Additionally, an increase in hull powder leads to a rise in porosity, from 31.25 to 43.89%. This is due to the formation of voids resulting from incomplete combustion of organic matter. While this porosity may improve thermal insulation, it compromises mechanical strength. Sutcu et al. report comparable results, with porosity reaching 50% for bricks incorporating plant residues. Overall, the data indicate an inverse correlation between the amount of palm kernel shell powder in the composite and its mechanical performance. However, the data also show an improvement in thermal properties and lightness. The best balance appears to be achieved with a palm kernel shell powder proportion of 15–25%, which maintains adequate strength and favorable porosity for insulation. These results are consistent with those of other researchers who have tested similar materials^43^.

### Limitations and future perspectives

Despite the rigorous methodology applied in this study, several limitations should be acknowledged to guide future research.

First, the physical, thermal, and chemical properties were analyzed comprehensively. However, the lack of detailed statistical treatment, such as standard deviations or significance tests, limits the robustness of comparisons between different formulations. Incorporating statistical tools would strengthen the validity of the observed mechanical performance trends.

Second, the discussion of how this study compares with existing literature is relatively brief. A more critical analysis of the discrepancies and similarities with related studies would provide a better context for this material within alternative construction solutions. Additionally, although palm kernel shell powder was tested at various proportions up to 60%, a significant decrease in compressive strength was observed at contents greater than 25%. However, no optimization strategy was proposed. Future investigations could explore adding natural binders, such as lime or gypsum, or pretreatment methods to improve interfacial bonding with the clay matrix. Finally, although thermal and chemical analyses identified key transformation stages (e.g., dehydroxylation and cellulose degradation), the direct correlation between these stages and macroscopic mechanical and thermal behavior could be further explored. Thermo-mechanical modeling or in situ tracking of structural evolution would provide more insight into the material’s performance. In summary, this study establishes a solid foundation for the use of agricultural waste in construction materials. However, further research is needed to optimize performance and promote its use in sustainable building practices.

## Conclusion

The goal of these studies was to synthesize sustainable, economical, and ecological materials. To achieve this objective, seven blends were developed, incorporating between 0% and 60% palm kernel shell powder. Each sample underwent appropriate physical, chemical, mechanical, and thermal characterizations.

First, the results of the physical characterization showed a progressive decrease in density with an increase in palm kernel shell powder content. The density decreased from 1801.45 kg/m^2^ for A0% to 1060.73 kg/m^2^ for A60%. This reduction in density is advantageous for constructions requiring lightweight materials, such as partitions or multistory elevations.

Secondly, in mechanical terms, compressive strength ranged from 4.64 MPa (A0%) to 0.12 MPa (A60%), indicating lower resistance to compression and an effective usage limit of around 25% palm kernel shell powder. Meanwhile, the A15% formulation offers a good compromise at 2.99 MPa, which is sufficient for certain non-load-bearing applications.

Furthermore, thermal and chemical analyses using TGA, DTG, and DTA revealed that formulations containing high amounts of palm kernel shell powder experience significant thermal mass loss (up to 80.03% for A60%), which is linked to the degradation of lignocellulosic components. DTG peaks between 313 and 463 °C reflect the thermal decomposition of cellulose and lignin. DTA curves show an increase in exothermic reactions with the addition of palm kernel shell powder. Spectroscopic (FTIR) and imaging (SEM-EDX) techniques confirmed the presence of functional organic groups and increased porosity linked to palm kernel shell powder. Overall, the characterization results confirm the material’s potential for sustainable construction applications. Using locally available resources combined with the material’s physical and thermal properties supports its viability in ecological and cost-effective building strategies.

## Data Availability

the datasets used and/or analysed during the current study are available from the corresponding author upon reasonable request.
